# Spoilage of Microfiltered and Pasteurized Extended Shelf Life Milk Is Mainly Induced by Psychrotolerant Spore-Forming Bacteria that often Originate from Recontamination

**DOI:** 10.3389/fmicb.2017.00135

**Published:** 2017-01-31

**Authors:** Etienne V. Doll, Siegfried Scherer, Mareike Wenning

**Affiliations:** Chair of Microbial Ecology, Institute for Food and Health, Technische Universität MünchenFreising, Germany

**Keywords:** microfiltration, extended shelf life (ESL) milk, microbiota, spoilage, psychrotolerant spore-forming bacteria

## Abstract

Premature spoilage and varying product quality due to microbial contamination still constitute major problems in the production of microfiltered and pasteurized extended shelf life (ESL) milk. Spoilage-associated bacteria may enter the product either as part of the raw milk microbiota or as recontaminants in the dairy plant. To identify spoilage-inducing bacteria and their routes of entry, we analyzed end products for their predominant microbiota as well as the prevalence and biodiversity of psychrotolerant spores in bulk tank milk. Process analyses were performed to determine the removal of psychrotolerant spores at each production step. To detect transmission and recontamination events, strain typing was conducted with isolates obtained from all process stages. Microbial counts in 287 ESL milk packages at the end of shelf life were highly diverse ranging from <1 to 7.9 log cfu/mL. In total, 15% of samples were spoiled. High G+C Gram-positive bacteria were the most abundant taxonomic group, but were responsible for only 31% of spoilage. In contrast, psychrotolerant spores were isolated from 55% of spoiled packages. In 90% of samples with pure cultures of *Bacillus cereus* sensu lato and *Paenibacillus* spp., counts exceeded 6 log cfu/mL. In bulk tank milk, the concentration of psychrotolerant spores was low, accounting for merely 0.5 ± 0.8 MPN/mL. *Paenibacillus amylolyticus/xylanexedens* was by far the most dominant species in bulk tank milk (48% of all isolates), but was never detected in ESL milk, pointing to efficient removal during manufacturing. Six large-scale process analyses confirmed a high removal rate for psychrotolerant spores (reduction by nearly 4 log-units). *B. cereus* sensu lato, on the contrary, was frequently found in spoiled end products, but was rarely detected in bulk tank milk. Due to low counts in bulk tank samples and efficient spore removal during production, we suggest that shelf life is influenced only to a minor extent by raw-milk-associated factors. In contrast, recontamination with spores, particularly from the *B. cereus* complex, seems to occur. To enhance milk quality throughout the entire shelf life, improved plant sanitation and disinfection that target the elimination of spores are necessary.

## Introduction

Extended shelf life (ESL) products have largely replaced conventionally pasteurized milk due to the growing demand for fluid milk products with prolonged shelf life. Technologies for producing ESL milk include high-heat treatment, high hydrostatic pressure (Datta and Deeth, 1999; Chawla et al., 2011), pulsed electric fields (Walkling-Ribeiro et al., 2011), bactofugation (Te Giffel and Van Der Horst, 2004), or microfiltration (Fernandez Garcia et al., 2013). The most commonly used technique in Germany is high-heat treatment, followed by microfiltration. To the authors’ knowledge, high hydrostatic pressure and pulsed electric fields are currently not used in commercial production of ESL milk.

Combined microfiltration (MF) and pasteurization enables ESL milk production with only minor thermal treatment compared to that of ultra-high temperature (UHT) processed milk. Organoleptic properties similar to conventionally pasteurized milk are therefore maintained and promote broad consumer acceptance. In the commercial Bactocatch procedure originally proposed by Holm et al. (1989), raw milk is separated into skim milk and a cream fraction. Skim milk is filtered through ceramic membranes with a nominal pore size of 1.4 μm. Containing a large proportion of microorganisms, the retentate is then either removed or combined with the cream fraction and UHT treated (135–150°C, 2–3 s). After adjusting the fat content, the milk is pasteurized to inactivate remaining vegetative cells. The entire process reduces the bacterial load by a total of 4.6–5.6 log-units. Depending on the storage temperature, shelf lives of 22–29 days at 8°C and even 26–33 days at 6°C are achieved (Elwell and Barbano, 2006; Schmidt et al., 2012; Caplan and Barbano, 2013; Fernández García and Riera Rodríguez, 2014).

The quality of ESL milk at the end of shelf life, however, is subject to strong fluctuations and premature spoilage constitutes a considerable problem. In a study of Schmidt et al. (2012), bacterial counts ranged in between <1 and 8 log cfu/mL and 8% of all analyzed retail packages were already spoiled at the expiration date. Analyses of predominant species revealed that two microbial groups were responsible for premature spoilage: Gram-negative post-pasteurization recontaminants (PPR) and psychrotolerant spore-forming bacteria (PSF), mainly *Bacillus cereus* sensu lato and *Paenibacillus* spp. (Schmidt et al., 2012). This is in line with findings for conventionally pasteurized milk, where PSF are the limiting factor for shelf life as soon as PPR with Gram-negative organisms is sufficiently controlled (Fromm and Boor, 2004; Huck et al., 2008).

To avoid premature spoilage of ESL milk, it is essential to understand and control all potential routes along which PSF may enter the product: as part of the raw milk microbiota or as recontaminants in the dairy plant. The mild conditions of pasteurization do not inactivate spores. Consequently, spore-forming bacteria in conventionally pasteurized milk mainly originate from raw milk and are transmitted through the entire process (Huck et al., 2007; Bartoszewicz et al., 2008). However, in conventionally pasteurized milk recontamination with spore-forming bacteria was found to occur at different stages of processing (Svensson et al., 1999, 2000; Eneroth et al., 2001; Salustiano et al., 2009; Kumari and Sarkar, 2016). The entry points for ESL milk are not clear yet; however, microfiltration reduces spore transmission from raw milk. Data concerning the removal of PSF that are naturally present in raw milk is not available to date, but retention rates achieved in spiking experiments ranged between 2.0 and 4.5 log-units (Trouvé et al., 1991; te Giffel et al., 2006; Tomasula et al., 2011). Their small size prevents the complete removal of spores, which makes them an inherent problem.

In this study, we aimed to extend knowledge about the dominant microbiota and spoilage-inducing organisms in microfiltered and pasteurized ESL milk. We also assessed the origin of PSF and possible routes of transmission along the production chain.

## Materials and Methods

The study consists of three parts (Supplementary Figure S1): (i) analyzing end products, by determining total aerobic bacterial counts (TACs) and predominant microbiota at the end of shelf life; (ii) analyzing bulk tank milk, including counts of mesophilic and psychrotolerant spores, as well as biodiversity of psychrotolerant spores; and (iii) process analysis addressing the removal of psychrotolerant spores along the production chain. We compared isolates obtained from different process stages using species affiliation and strain typing to ultimately investigate possible transmission and recontamination. For each batch of end products, the corresponding bulk tank milk was obtained, stored, and partially used for further analysis. We also included samples of bulk tank milk as well as end products of process analyses in other parts of the study.

### Assessment of Microbial Counts and Dominant Species in ESL Milk

To assess the dominant microbiota and bacterial counts in ESL milk, a total of 287 retail packages were analyzed at the end of shelf life. The samples represented 39 batches (different production dates) from four different dairies and included conventional (*n* = 103) and organic (*n* = 184) milk with fat contents of 1.5% (*n* = 117), 3.5% (*n* = 151), and 3.8% (*n* = 19). Of each production batch, several packages containing 1 L ESL milk were analyzed. We examined 111 packages of 14 batches for dairy A, 53 packages of nine batches for dairy B, 74 packages of nine batches for dairy C, and 49 packages of seven batches for dairy D. End products and the corresponding bulk tank milk were obtained for 33 batches (236 packages; Supplementary Figure S1). The end product was analyzed immediately; the bulk-tank-milk samples were stored at -20°C and analyzed later for PSF counts (see Determination of Mesophilic and Psychrotolerant Spore Counts in Bulk Tank Milk). Six batches of end products (51 packages) originated from process analyses described in part Section “Identification of Bacterial Isolates.”

The milk was shipped cooled to between 6 and 8°C via overnight express; end products were incubated at the recommended storage temperature of 8°C until end of shelf life. Each package’s TAC and microbial composition were subsequently analyzed. Serial dilutions were prepared in Ringer solution, plated on tryptic soy agar (TSA; Roth, Karlsruhe, Germany) in duplicates, and incubated for 5 days at 30°C. Resulting colonies were counted and the relative abundance of each distinct colony morphology was estimated. The four to seven most dominant morphologies were subcultured on TSA to determine each package’s predominant microbiota. All resulting pure cultures (*n* = 1590) were identified using FTIR spectroscopy and representative isolates of PSF were further identified by 16S rDNA or *rpoB* sequence analyses.

### Determination of Mesophilic and Psychrotolerant Spore Counts in Bulk Tank Milk

A total of 360 bulk-tank-milk samples were obtained from the bulk tanks of 12 dairies, including dairies A–D, and from an additional eight dairies that do not produce ESL milk. The milk was shipped cooled via overnight express and analyzed directly upon arrival at our laboratory or after refrigeration at -20°C. To assess seasonal influences on spore counts, the samples were collected over a time span from February 2014 to January 2016. Between 5 and 23 samples were analyzed each month.

To enumerate mesophilic spores, milk samples were held at 80°C for 10 min and plated on TSA in duplicates. Colonies were counted after incubation at 30°C for 2 days (*n* = 354). Due to their low concentration in raw milk, psychrotolerant spores were enumerated using the five-tube most probable number (MPN) method described by McGuiggan et al. (2002) with several modifications (*n* = 360). The bulk-tank-milk sample was thoroughly mixed and split into five aliquots of 10, 1, and 0.1 mL each. The final volume of all tubes was set to 10 mL with tryptic soy broth (TSB; Merck, Darmstadt, Germany). An additional sterile tube with 10 mL TSB was included as negative control. The samples were held at 80°C for 10 min and subsequently cooled down in ice water. 100 μL of L-alanine (Merck, Darmstadt, Germany; 10% w/v in phosphate buffer 0.01 M, pH 7.2) were added as germination agent and all tubes were incubated at 6°C for 21 days to allow outgrowth and hence the detection of all psychrotolerant spores present. Afterward, one loopful of each tube was streaked on TSA and plates were incubated at 30°C for 2 days. Tubes were counted positive if at least five colonies of identical morphology were present. The spore counts were subsequently determined using MPN tables (Harrigan and McCance, 1979) taking into account the number of tubes positive for psychrotolerant growth.

### Analysis of Biodiversity of Psychrotolerant Spores in Bulk Tank Milk

The biodiversity of psychrotolerant spores was assessed in 28 samples from all three parts of this study (Supplementary Figure S1). Eighteen samples were chosen to analyze the general biodiversity of PSF in bulk tank milk with varying spore counts (0.02–16 MPN/mL) and from different seasons. Six samples originated from process analyses, for which the biodiversity had already been determined (see Identification of Bacterial Isolates). To assess whether spoilage-inducing PSF were transmitted from raw to ESL milk, four additional samples were analyzed after end products of the same batch tested positive for the growth of psychrotolerant spores. The protocol was identical for all setups, differing only in the desired number of isolates and hence the number of inoculated test tubes. All bulk-tank-milk samples were first analyzed for their MPN count. The bulk tank milk was then divided into 100 (process analyses), 160 (general biodiversity), or 300 aliquots (bulk tank milk of positive end products) containing theoretically 0.9 spores and enriched with 7 mL TSB. All samples were held at 80°C for 10 min and 100 μL of L-alanine (10% w/v) were added. After incubation at 6°C for 21 days, one loopful of each tube was streaked on TSA and incubated at 30°C for 2 days. Isolates showing growth at refrigerated temperatures, as indicated by at least five morphologically identical colonies on one plate, were then subcultured on TSA. The resulting pure cultures were identified by FTIR spectroscopy and representative bacteria were further identified by their 16S rDNA and *rpoB* gene sequence. Selected isolates were also typed at strain level.

### Process Analysis of Microfiltered and Pasteurized ESL Milk

In six large-scale process analyses, the efficiency of combined MF and pasteurization for removing psychrotolerant spores was determined and isolates of PSF were obtained for subsequent strain typing. Three productions each of dairy A (A) and dairy B (B) were analyzed. The milk was treated with the Bactocatch procedure: raw milk was degreased and skim milk was filtered through ceramic membranes with a nominal pore size of 1.4 μm. After adjusting the fat content to 1.5% (*n* = 5) or 3.5% (*n* = 1) with UHT-treated cream (B) or UHT-treated cream-retentate mixture (A), the milk was pasteurized and filled. Counts of psychrotolerant spores were determined in bulk tank milk, skimmed milk, permeate, and pasteurized milk and all isolates were identified. Samples that were subjected to UHT treatment were not analyzed further. Large sample volumes were used due to low spore concentrations in bulk tank milk and expected high removal rates during processing. The following aliquots were prepared: 100 x 1 mL (A) or 2 mL (B) of bulk tank milk enriched with 7 mL of TSB, 100 x (A) or 150 x (B) 10 mL of skimmed milk, 75 × 150 mL (A) or 90 × 200 mL (B) each of permeate and pasteurized milk. Bulk tank milk, skimmed milk, and permeate were held at 80°C for 10 min to inactivate vegetative cells and all samples including the pasteurized milk that was not subjected to further heat-treatment were incubated at 6°C for 21 days. One loopful of each tube or flask was subsequently plated on TSA and the plates were incubated at 30°C for 2 days. Spore counts were then determined taking into account the number of units positive for bacterial growth relative to the total sample volume and all isolates were identified by FTIR spectroscopy and 16S rDNA or *rpoB* gene sequencing. Microbial counts and the dominating species in the resulting end products (*n* = 51) were also analyzed at the end of shelf life as described in Section “Assessment of Microbial Counts and Dominant Species in ESL Milk.” For determination of possible transmission of or recontamination by PSF along the production chain, strain typing was performed with selected isolates originating from different process steps.

### Identification of Bacterial Isolates

All isolates obtained in this study were identified using FTIR spectroscopy (Oberreuter et al., 2002; Wenning et al., 2014; von Neubeck et al., 2015). The strains were cultured for 24 ± 0.5 h under the following conditions: spore-forming bacteria on TSA (Oxoid, Wesel, Germany) at 25°C, lactic acid bacteria on All Purpose Tween agar (APT; Merck, Darmstadt, Germany) at 34°C anaerobically, and all other isolates on TSA at 30°C. One loopful of the resulting confluent lawn was suspended in 100 μL of sterile water and 25 μL of the homogenous suspension were transferred to a 96-well zinc selenide sample carrier. The samples were then dried at 42°C for 45 min to form a continuous film. FTIR spectra were then recorded by a Tensor 27 spectrometer coupled to the HTS-XT device for high sample throughput (both from Bruker Optics, Ettlingen, Germany) and evaluated as described by Oberreuter et al. (2002). Data analysis was performed using OPUS software V7.2 (Bruker Optics, Ettlingen, Germany). Three in-house databases containing approximately 8000 spectra of 240 genera and 1000 species were used to identify isolates.

Representative isolates of psychrotolerant spores were also identified by their 16S rDNA or, if they belonged to the genus *Paenibacillus*, using the more discriminative *rpoB* gene sequence. Isolates were selected separately for each milk sample using hierarchical cluster analysis (HCA) of the FTIR spectra (Wenning and Scherer, 2013). At least one isolate from each cluster was selected for sequencing. The sequencing result was then extrapolated to all isolates of the initial cluster to obtain the actual abundance of all identified species.

Cell lysis and 16S rDNA amplification were performed as described by von Neubeck et al. (2015), with primers 27f (5′-AGAGTTTGATCCTGGCTCA-3′) and 1492r (5′-CGGCTACCTTGTTACGAC-3′). For amplification of the *rpoB* gene, primers StreptoF (5′-AARYTIGGMCTGAAGAAAT-3′) and StreptoR (5′-TGIARTTTRTCATCAACCATGTG-3′) were used, resulting in a 740 bp fragment (Drancourt et al., 2004). Cycling conditions were modified based on the protocol of Durak et al. (2006) with 20 cycles of touchdown PCR consisting of a denaturation step at 95°C for 30 s, annealing from 60 to 50°C for 30 s with temperature decrease of 0.5°C per cycle and elongation at 72°C for 1 min, 30 cycles of denaturation at 95°C for 1 min, annealing at 50°C for 30 s and elongation at 72°C for 1 min, with a final elongation at 72°C for 7 min. Sequencing was performed by LGC Genomics (Berlin, Germany), with primers 926r (5′-CCGTCAATTCTTTGAGTTT-3′) or 1061r (5′-CRRCACGAGCTGACGAC-3′) for 16S rDNA and StreptoF for *rpoB* gene sequences. Identification was carried out using the EzTaxon server for 16S rDNA (Kim et al., 2012) and the BLAST algorithm for *rpoB* data (Altschul et al., 1990). For species allocation of *Paenibacillus* isolates, we established a database containing the *rpoB* gene sequences of 44 species that are commonly found in milk, including 12 type strains. Non-type strain *rpoB* sequences were obtained from NCBI or previously identified isolates. Similarity cutoffs for species and genus demarcation were set at 98.65 and 95% sequence identity for 16S rDNA (Kim et al., 2014) and for species affiliation using the *Paenibacillus* specific *rpoB* sequence at 95% identity. All isolates with similarities below the cutoff value were classified as potential novel species or genera. To confirm phylogenetic allocation, their sequences were aligned with (type) strains of closely related species using MEGA version 6 (Tamura et al., 2013). All presumptive novel species and genera were numbered consecutively from most to least abundant.

### Strain Typing of Psychrotolerant Spore-Forming Bacteria

To detect possible transmission or recontamination with psychrotolerant spores along the production chain, isolates obtained from different process stages were further characterized and compared at strain level. Included were isolates from all productions with end products containing PSF as well as process analyses where PSF were detected in more than one of the process steps (Supplementary Figure S1). In total, 10 batches were included and candidate strains for typing were selected using HCA. The relatedness of all representative psychrotolerant spores was determined for each of the 10 batches. If FTIR spectra from isolates of the same species obtained from different process steps clustered closely together, they were considered to be clones and further analyzed by randomly amplified polymorphic DNA (RAPD)-PCR. To capture additional matching strains that did not cluster together, several isolates of identical species from different process steps and *B. cereus* sensu lato as well as *Paenibacillus odorifer* strains from different batches obtained from the same dairy were included in further strain typing. In total, 90 isolates were selected.

Cell lysis for all strains was performed as described previously (von Neubeck et al., 2015). The DNA concentration of cell lysates was determined spectrophotometrically with NanoDrop^®^ ND-1000 (Peqlab, Darmstadt, Germany) and adjusted to 50 ng/μL. Three different primers were used for RAPD analyses that had proven to be very discriminative for sporeformers in previous experiments. OPA7 (5′-GAAACGGGTG-3′) and N5 (5′-CGGCCACTGT-3′) (Nilsson et al., 1998) were used for every isolate and primer OPB18 (5′-CCACAGCAGT-3′) (Woodburn et al., 1995) was only used if with one of the first primers failed to produce a clear band pattern. Amplification was performed with a KAPA2G^TM^ Robust Hot Start DNA Polymerase Kit (Peqlab, Darmstadt, Germany). For each reaction, 5 μL Enhancer, 5 μL BufferA, 0.5 μL dNTPs (10 mM), 2 μL primer (50 μM), 0.1 μL DNA polymerase, and 1 μL lysate were used. The following reaction conditions were used: 30 cycles of denaturation at 94°C for 30 s, annealing at 30°C (N5), 32°C (OPA7), or 35°C (OPB18) for 40 s and elongation at 72°C for 3 min with an initial denaturation step of 94°C for 3 min and final elongation at 72°C for 3 min. Another strain belonging to the species of interest was also included as outgroup. Amplification products were visualized on 2% agarose gels after gel electrophoresis at 150 V for 2 h in 0.5x TBE buffer. As the primers chosen were very discriminative and band patterns obtained differed largely in band number and band intensity (Supplementary Figure S2), they were evaluated visually.

### Statistical Analyses

Pearson’s chi-squared test was used to test the association (i) between TAC of end products at the end of shelf life (<6 and ≥6 log cfu/mL) and prevalence of high G+C Gram-positive bacteria, Gram-negative bacteria and PSF and (ii) of the occurrence of bacterial species in bulk tank milk and ESL milk. To determine seasonal influences on counts of mesophilic and psychrotolerant spores in bulk tank milk, the concentrations determined in each month were tested for differences in central tendencies by the Kruskal–Wallis test. Pairwise comparison of months was then conducted using the Wilcoxon Rank Sum test. All tests were performed using R version 3.1.2. *p* < 0.05 was considered statistically significant and *p* < 0.01 highly significant.

## Results

### Microbial Composition of ESL Milk at the End of Shelf Life

**FIGURE 1:**
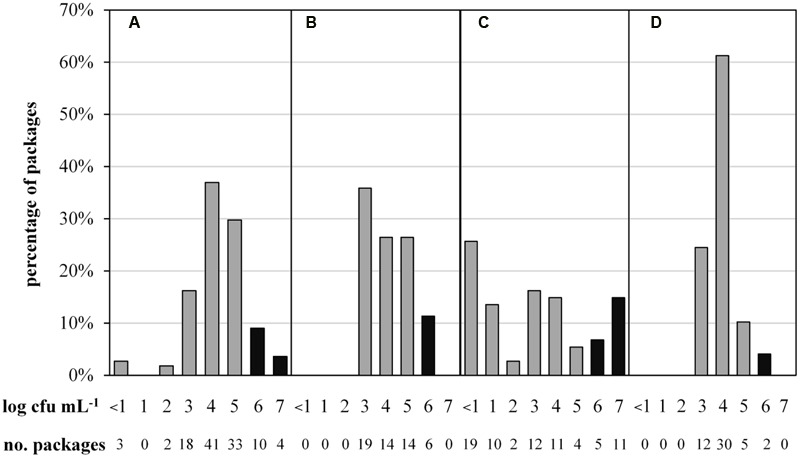
**Abundance distribution of aerobic bacterial counts in 287 packages of microfiltered and pasteurized ESL milk at the end of shelf life after storage at 8°C.** Samples were obtained from for four different dairies (**A**, *n* = 111; **B**, *n* = 53; **C**, *n* = 74; **D**, *n* = 49). Black bars indicate spoilage.

To determine the microbial status of ESL milk, TAC and the predominant microbiota of 287 retail packages from four dairies were analyzed at the end of shelf life. The maximal TAC in fresh milk is unregulated in Germany, but 6 log cfu/mL is generally regarded as the limit for bacterial spoilage and was also applied in this study. Bacterial counts were highly diverse, ranging from <1.0 to 7.9 log cfu/mL. Even within single batches, TAC varied up to three log-units per mL. The majority of packages showed acceptable microbial counts, containing 3 to 5 log cfu/mL and 13% had very low TAC with as little as <1 to 2 log cfu/mL. However, a total of 15% of packages were spoiled with ≥6.0 log cfu/mL. Considerable discrepancies were observed between different dairies (**Figure 1**). Dairy C not only had the highest proportion of spoiled packages but also the largest spread of TAC and the highest proportion of packages with very low microbial counts. Dairy D’s products exhibited the least differences in TAC and the lowest proportion of spoiled end products.

**FIGURE 2:**
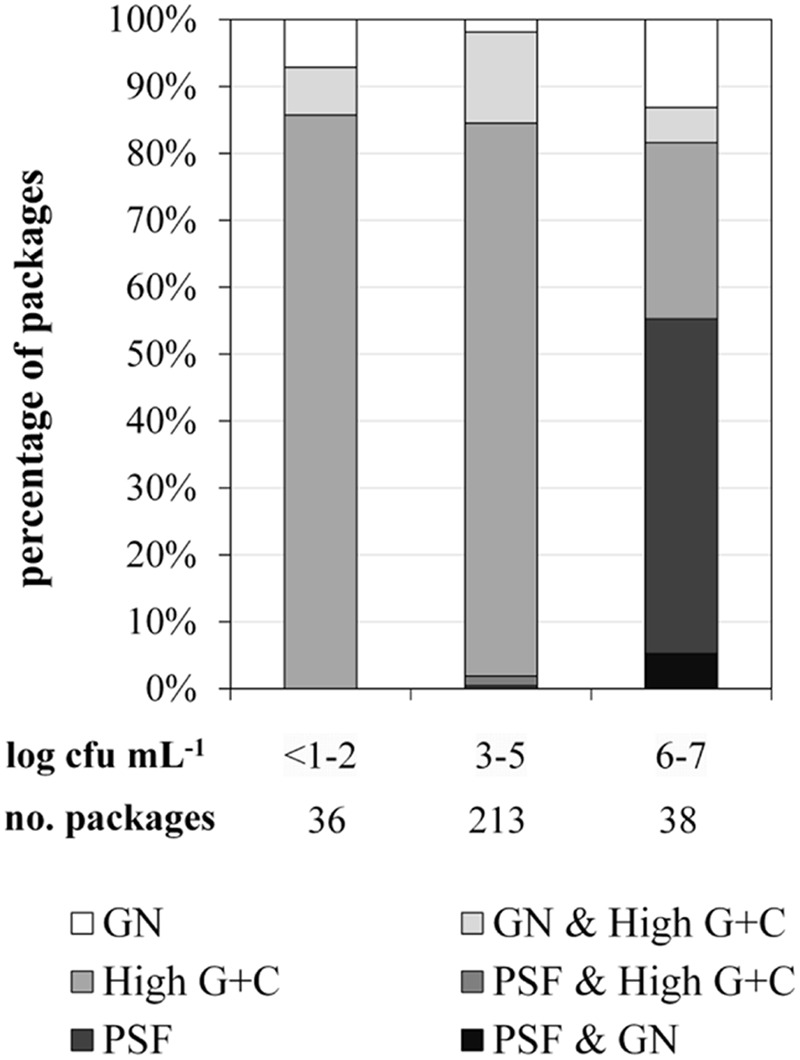
**Distribution of predominant bacterial groups in 287 packages of microfiltered and pasteurized ESL milk at the end of shelf life after storage at 8°C in correlation to aerobic bacterial counts.** GN, Gram-negative bacteria; PSF, psychrotolerant spore-forming bacteria.

For FTIR-spectroscopy-based analyses of the predominant ESL milk microbiota, a total of 1590 isolates obtained from 287 retail packages were identified. Three bacterial groups were detected: high G+C Gram-positive microorganisms such as *Microbacterium*, Gram-negative recontaminants (e.g., *Moraxella* and *Pseudomonas*), and PSF (*Bacillus* and *Paenibacillus* spp.). The microbiota of packages with TAC ranging in between <1.0 and 5.9 log cfu/mL was clearly dominated by Gram-positive high G+C bacteria (**Figure 2**). They constituted the predominant group in more than 90% of ESL milk samples regardless whether they had an exceptionally low TAC or were almost spoiled. However, in spoiled packages with TAC >6 log cfu/mL there was a highly significant shift of the microbial composition (*p* < 0.01). The abundance of Gram-positive high G+C bacteria decreased to only 30% whereas 50% of the packages contained exclusively psychrotolerant spores and another 5% a mixed flora of psychrotolerant spores and Gram-negative recontaminants (**Figure 2**). The percentage of samples dominated by Gram-negative recontaminants (alone or in combination with high G+C Gram-positive bacteria) was found to range between 15 and 20% regardless of TAC. Again, there were remarkable discrepancies between different dairies. Spoiled packages from dairies B and D contained exclusively Gram-positive high G+C bacteria. Among samples from dairy C, only 20% were spoiled by this group and the remaining 80% contained PSF. Spoiled packages from dairy A were dominated by all three groups with Gram-negative recontaminants having the highest proportion.

To elucidate the risk for milk spoilage associated with each individual group of organisms, we assessed the frequency of spoilage within packages that contained predominantly one of the bacterial groups (mixed populations excluded; **Table 1**). Whereas high G+C Gram-positive bacteria were dominant in 203 packages (71%), only 6% of these samples were spoiled. Gram-negative bacteria were found to be predominant in merely 10 packages (4%); however, half of these samples showed premature spoilage. The most important group was PSF. It was dominant in only 7% of milk samples but proliferated up to ≥6 log cfu/mL in 90% of these packages and thereby showed the greatest spoilage potential.

**Table 1:** Incidence of predominant bacterial groups in 234 packages of ESL milk at the end of shelf life after storage at 8°C and respective frequency of spoilage.

	No. of packages	Frequency of spoilage
Psychrotolerant spores	21	90%
Gram-negative recontaminants	10	50%
Gram-positive high G+C	203	5%

*Only packages containing exclusively one bacterial group are included.*

Because it constitutes the most important group of spoilage organisms in microfiltered ESL milk, the prevalence of different PSF species was further analyzed (Supplementary Figure S3). A total of 25 packages from seven batches of two different dairies contained psychrotolerant spores as the predominant bacterial group. To avoid an overestimation, each species was counted only once per positive package. This resulted in 36 representative isolates belonging to nine different species of the genera *Paenibacillus* and *Bacillus*. *B. cereus* sensu lato was by far the most abundant species group. It represented 36% of psychrotolerant spores in ESL milk and was detected in 13 packages from four different batches, corresponding to more than half of all units testing positive. Other abundant species were *P. odorifer* (33%), which was isolated from 12 packages from three batches and, interestingly, a presumptive novel species *Paenibacillus* sp. nov. 1 (6%). The latter, however, was found in only one batch. All other species of psychrotolerant spores were isolated from only one or two packages and are therefore of minor relevance.

### Prevalence and Biodiversity of Psychrotolerant Spore-Forming Bacteria in Bulk Tank Milk

**FIGURE 3:**
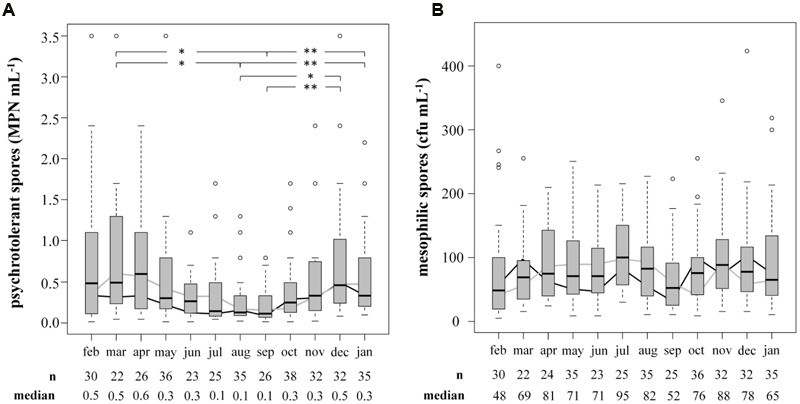
**Influence of the season on counts of psychrotolerant (A)** and mesophilic **(B)** spores in bulk tank milk. Two consecutive years from February 2014 to January 2016 were analyzed. Boxplots display the combined values of both years. The boxes represent first quartile, median, and third quartile, the whiskers represent mild outliers (1.5 x interquartile range), and open circles represent more extreme outliers. Y axes are discontinued at 3.5 MPN mL^-1^ for psychrotolerant spores and at 400 cfu mL^-1^ for mesophilic spores. The following outliers are not displayed. Psychotolerant spores: Feb: 9.2; Apr: 5.4; May: 5.4, >16.0; Oct: >16.0; Nov: 9.2; Mesophilic spores: Jan: 468; Aug: 4,073; Oct: 2,300; Dec: 896. black line: geometric mean in year one; gray line: geometric mean in year two.^∗^*p* < 0.05; ^∗∗^*p* < 0.01.

The prevalence and biodiversity of psychrotolerant spores were investigated to evaluate the importance of bulk tank milk as a potential source of them in ESL milk. Accounting for only 0.57 ± 0.80 MPN/mL, the concentration of psychrotolerant spores in bulk tank milk (*n* = 360) was low. In comparison, mesophilic spores accounted for 90 ± 80 cfu/mL (*n* = 354), reaching a 200-fold higher concentration. To evaluate seasonal influences on spore counts, samples were obtained over 2 years and an average of 15 samples was analyzed each month (**Figure 3**). We found the concentration of psychrotolerant spores in summer and early autumn (August and September) to be significantly lower than that in other months (March, December, January; *p* < 0.05). The median from July to September ranged in between 0.10 and 0.14 MPN/mL. During winter and spring, the spore counts increased 3- to 4-fold and in April the maximum median of 0.59 MPN/mL was detected. This trend was confirmed in both years of analysis. Additionally, not only the concentration but also the variance of psychrotolerant spores between different samples decreased in summer. In contrast, the counts of mesophilic spores showed no seasonality.

For biodiversity analyses, a total of 2634 isolates obtained from 28 bulk-tank-milk samples were identified by FTIR spectroscopy and 875 representative isolates were further identified by their 16S or *rpoB* gene sequence. Overall, a richness of 53 different species belonging to nine genera was detected (Supplementary Table S1). The two most abundant genera were *Paenibacillus* and *Bacillus*. They accounted for 80 and 10% of isolates respectively and were represented by 27 and 9 different species. At species level, *Paenibacillus amylolyticus/xylanexedens* was clearly dominant. Remarkably, it was isolated 1250 times and thereby represented almost half of all isolates (48%). Other abundant species were *P. odorifer* (10%), *Paenibacillus taichungensis/tundrae* (8%), the presumptive novel species *Paenibacillus* sp. nov. 1 (7%), and *Bacillus pumilus/safensis* (7%). Most of the species, however, were rare and occurred with fractions of at most 3% each. The 35 least abundant species together accounted for as little as 3.5% of isolates, confirming an uneven abundance distribution. Among the 2634 isolates, there was a considerable number of species and genera that displayed only low similarity of <98.65% for 16S rDNA and 95% for *rpoB* gene sequences to known species. Some strains had a similarity of <95% for 16S rDNA. Although no additional experiments were carried out to confirm the distinctness of those isolates from known species and genera, the genetic data give a strong indication that they belong to hitherto unknown species. In total, 18 potential novel species and three novel genera were detected, which together made up 14% of all isolates. A remarkably great abundance was found for two of the novel species that were assigned to the genus *Paenibacillus*. They represented respectively 6.6 and 3.2% of isolates.

### Spore Removal during Processing of ESL Milk

The efficiency of combined MF and pasteurization for removing psychrotolerant spores was analyzed during the production of six batches of organic ESL milk from two dairies. Samples were taken at five different points throughout processing: bulk tank milk, after separation of cream, after MF, after pasteurization, and after filling.

**FIGURE 4:**
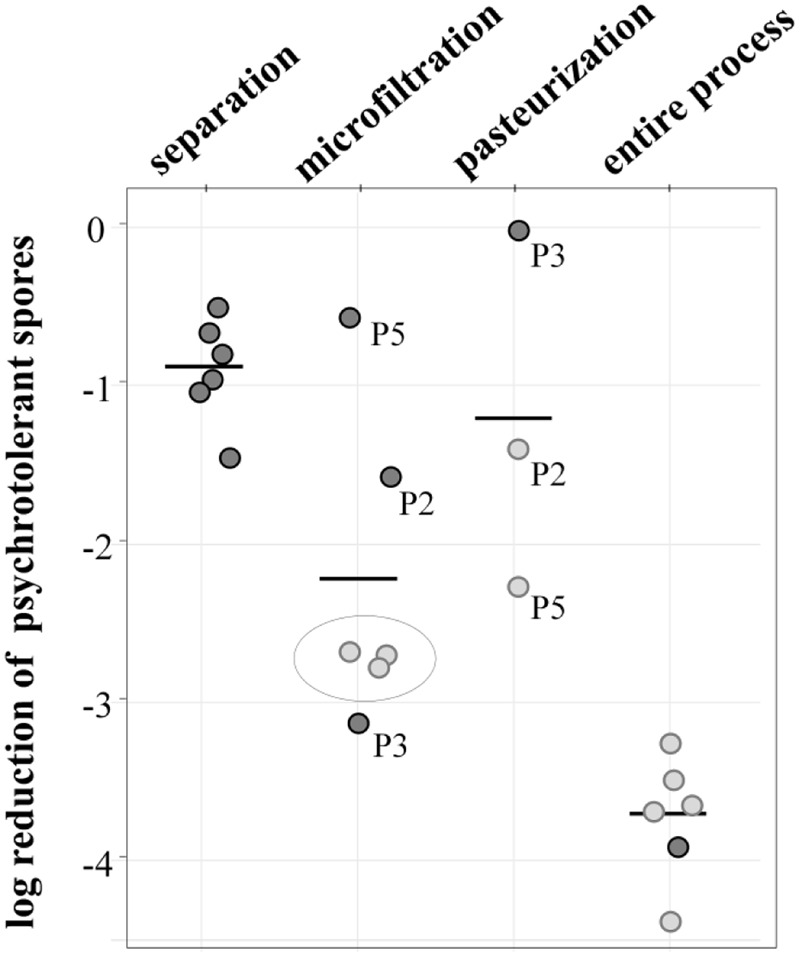
**log reduction of psychrotolerant spores achieved by processing six batches of microfiltered and pasteurized ESL milk.** Dark gray: decimal reduction of psychrotolerant spores as achieved by the respective process step; light gray: minimal reduction of psychrotolerant spores where actual removal could not be assessed due to spore counts below detection limits of 0.06 or 0.08 L^-1^. The values display the log reduction from previous process step to the detection limit and are therefore an underestimation of the actual removal; gray circle: spore counts in permeate below detection limit—in these process analyses, the efficiency of pasteurization could not be assessed.

Overall, the process achieved a potent reduction of 3.7 log-units (**Figure 4**). The initial separation of cream already led to a decrease of 0.9 log-units, but the largest removal rate was attained during MF, accounting for 2.2 log-units on average. Final pasteurization achieved an additional 1.2 log reduction. However, the available data show differences for independent runs, especially for MF. Here, the values for decimal reduction of psychrotolerant spores ranged from 0.6 or 1.5 log-units in impaired processes, up to 3.1 log-units. Interestingly, we found pasteurization to be more efficient (-1.4 and -2.4 and log-units, respectively) after runs with dysfunctional MF (P2, P5) than after those with fully functional MF (no further removal in P3). This was unexpected, because pasteurization conditions are unable to inactivate spores. Underestimation of removal by MF may be one reason for the high effectiveness determined in the pasteurization step in process analysis P5 (**Figure 4**). Here, the permeate samples were taken during the first hour of the MF process. Retention of microbial cells in the beginning was possibly less efficient due to a lack of protein layer that becomes established during the first hour of MF and contributes to bacterial retention. In P2 and P3, pasteurized milk was sampled after 3–4 h.

The data generally has some limitations due to very low spore counts as indicated in **Figure 4**. Despite large sample volumes of up to 18 L, no spores were detected in several permeate and pasteurized milk samples, corresponding to counts of <0.06 or 0.08 MPN/L. In consequence, only minimal or in case of three pasteurizations, no reduction rates could be assessed. The given removal of psychrotolerant spores is therefore underestimated to a certain degree.

### Determination of Psychrotolerant Spore-Forming Bacteria’s Entry Points along the Processing Chain

**FIGURE 5:**
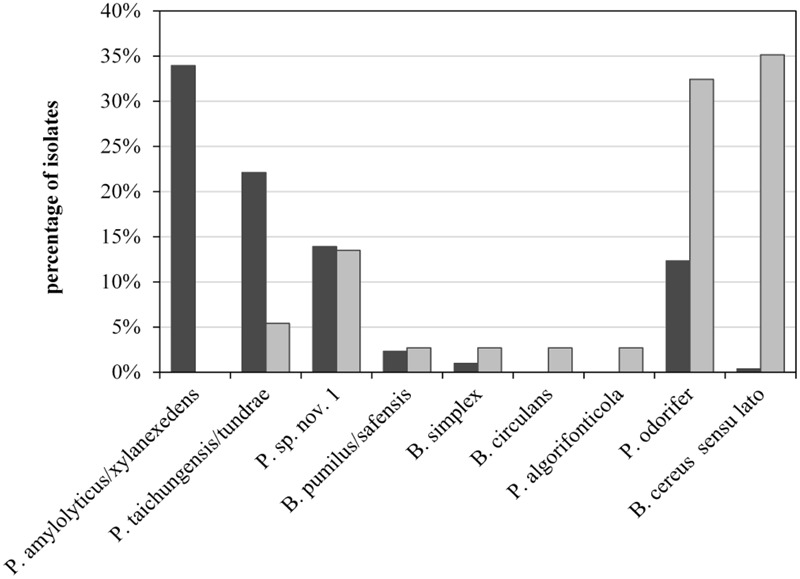
**Abundance of psychrotolerant spore-forming bacteria at species level obtained from bulk tank milk (black bars, *n* = 819) and corresponding end products (gray bars, *n* = 35) from six batches of microfiltered and pasteurized ESL milk**.

To assess the role of raw milk as a possible source of psychrotolerant spores in end products, we compared different production batches of previous analyses in detail. Seven of the 39 milk batches contained end products with PSF. For six of these, the corresponding bulk tank milk was available and had been analyzed for biodiversity of PSF. The species composition of bulk-tank-milk samples (819 isolates) and their corresponding end products (35 isolates) were initially compared. Conspicuous differences were discovered among the prevalences of all species found in the six ESL milk batches and their abundance in bulk tank milk (**Figure 5**). Whereas *P. amylolyticus/xylanexedens* was clearly the most dominant species in bulk tank milk, combined MF and pasteurization entirely removed it. Not even a single isolate was obtained from ESL milk. In contrast, *B. cereus* sensu lato represented merely 0.4% of bulk tank milk isolates but 35% of psychrotolerant spores in ESL milk. So the proportion of *B. cereus* sensu lato among the isolates increased almost 90-fold during milk processing. Also, *Bacillus circulans* and *Paenibacillus algorifonticola* were isolated exclusively from end products. For all these species, no association was found between the occurrence in bulk tank milk and that in end products (*p* < 0.01). Several other spore-forming bacteria (e.g., *P. odorifer, P. taichungensis/tundrae*, the presumptive novel species *Paenibacillus* sp. nov. 1, and *B. pumilus/safensis*) were detected to a similar degree in raw milk and end products. This was confirmed by the Pearson’s chi-squared test (*p* = 0.17).

To gain further insight into possible points of entry, RAPD typing was performed with 90 strains from different samples and process steps. The presence of identical strains at different stages of the production process provides evidence for the vertical transmission of microorganisms along the production chain. Missing consistencies between strains found in end products and different processing steps would point rather to horizontal transmission (recontamination events).

Transmission of psychrotolerant spores from bulk tank milk to later process stages was observed in 5 of 10 batches of ESL milk (**Table 2**), but psychrotolerant spores were found to reach the end product from only three batches (batches 1, 4, and 5). It is remarkable that this was attested in two cases for strains that were highly abundant in bulk tank milk: in batches 1 and 5, both having about 10 times higher spore counts in the bulk tank milk than other batches. In batches 2 and 3, the transmission did not extend to the final product, but terminated earlier. *P. odorifer* (batch 2) was found in bulk tank milk, skim milk, and permeate, but it was absent in the end products. As determined during process analysis, spores in this batch were inefficiently removed during the MF step (-0.6 log-units). However, at least partial underestimation of MF efficiency is likely, most probably due to early sampling at the very beginning of the production as already mentioned. Batch 3 constituted an exception. Here, the identical *B. cereus* sensu lato strain was detected in raw, skim, and pasteurized milk although it represented only the 5th most abundant species in bulk tank milk (5%) and the MF was very efficient (-3.1 log-units). Interestingly, in the same batch another *B. cereus* sensu lato strain was isolated exclusively from one end product and not from the bulk tank milk.

**Table 2:** Summary of all transmission events of psychrotolerant spore-forming (PSF) bacteria from bulk tank milk to later process stages as determined by RAPD typing of 90 strains from 10 batches of ESL milk.

Batch	Species	Isolated from	Bulk tank milk	
			Abundance of species [% of PSF]	PSF count [MPN/mL]
1	*P. taichungensis/tundrae*	BM SM PE EP	90%	1.84
2	*P. odorifer*	BM SM PE	5%	0.17
3	*B. cereus sensu lato*	BM PA	5%	0.63
4	*B. pumilus/safensis*	BM EP	5%	0.33
5	*P.* sp. nov. 1	BM EP	42%	1.70
	*P. odorifer*	BM EP	31%	

*In five batches, no transmission was observed (data not shown). For batches 1–3, isolates were obtained from bulk tank milk (BM), skim milk (SM), permeate (PE), pasteurized milk (PA), and end products (EP) — for batches 4 and 5, from bulk tank milk and end products only.*

A large fraction of all typing results yielded no match with other isolates. Identical strains in different batches from the same dairy would provide evidence for a persisting recontamination source, but all *B. cereus* sensu lato strains that were obtained from two batches from dairy A and *P. odorifer* strains obtained from two dairy-C batches showed unique RAPD profiles and could not be affiliated to the same strain. Remarkably, all 10 packages from one dairy-C batch contained *P. odorifer* and *B. cereus* sensu lato, but these were not detected in the corresponding bulk-tank-milk sample, pointing to a recontamination event.

## Discussion

### Bacterial Spoilage of ESL Milk

In Germany, shelf life of microfiltered and pasteurized ESL milk is generally regulated to a maximum of 24 days. Under storage at the recommended temperature of 8°C, the product should remain safe and palatable for the entire period. The results of this study, however, clearly demonstrate that microbial growth constitutes a major problem for maintaining the impeccable quality of ESL milk. Of 287 retail packages that were analyzed at the end of shelf life after storage at 8°C, a total of 15% were spoiled, containing 6 log cfu/mL up to almost 8 log cfu/mL. Overall, the microbial counts were higher than in a previous study conducted at our institute (Schmidt et al., 2012), but mainly with milk from different dairies. Here, about 50% of all packages had counts below 3 log cfu/mL. Yet, accounting for 8% of all samples, a large fraction was spoiled. Identifying and eliminating the bacteria contributing to premature spoilage is therefore important for increasing the quality of ESL milk during its entire shelf life.

Three bacterial groups were found to be relevant for microbial spoilage: Gram-negative recontaminants, high G+C Gram-positive bacteria, and PSF. Gram-negative bacteria were isolated from 15% of all packages and were predominant in 20% of spoiled samples, showing that PPR is still not optimally controlled. High G+C Gram-positives were responsible for 31% of premature spoilage. They were by far the most abundant group and isolated as only bacterial group from 203 ESL milk packages. Their spoilage potential however was low. Merely 5% of the positive samples were spoiled, revealing low growth rates at refrigeration temperature. This confirms the findings of Schmidt et al. (2012), who isolated *Microbacterium* spp. from 65% of packages that all retained TAC <6 log cfu/mL throughout the entire shelf life. The third group, PSF, was most relevant for the quality of ESL milk. Despite their relatively low abundance (7%), *Bacillus* spp. and *Paenibacillus* spp. were isolated from 55% of all spoiled samples. 90% of packages testing positive contained 6 to 7 log cfu/mL, confirming their ability to proliferate well under refrigerated conditions (Meer et al., 1991; Huck et al., 2007). The presence of psychrotolerant spores in milk packages thus implies a 90% risk of premature spoilage. Ranieri et al. (2012) stated that even one *Paenibacillus* spore is sufficient to induce spoilage during shelf life. The results are in line with Schmidt et al. (2012), who reported that *B. cereus* sensu lato and two *Paenibacillus* species were responsible for two thirds of spoiled packages. These two genera were also found to be most relevant for product spoilage in traditionally pasteurized milk, as soon as Gram-negative recontamination was avoided (Fromm and Boor, 2004; Huck et al., 2007, 2008). Reduction of PSF counts therefore seems to be an important step toward reducing the incidence of ESL milk spoilage. But unlike Gram-negative bacteria that always result from product recontamination, the source of spore-forming bacteria is much more difficult to determine. Since they are resistant to pasteurization, they are assumed to originate from bulk tank milk. However, contamination of milk in the dairy plant has been reported (Svensson et al., 1999; Eneroth et al., 2001).

### Counts and Biodiversity of Psychrotolerant Spore-Forming Bacteria in Bulk Tank Milk

Raw milk constitutes a potential source of PSF in ESL milk. The prevalence of PSF in bulk tank milk was assessed to gain insight into its role in the contamination process. The concentration of psychrotolerant spores was extremely low. They accounted for merely 0.57 MPN/mL, ranging between <0.02 MPN/mL and up to 16 MPN/mL in two outliers. These values accord with previous reports that found counts between 0.001 and 6.300 spores/mL (McKinnon and Pettipher, 1983; Meer et al., 1991; Mayr et al., 1999; McGuiggan et al., 2002; Masiello et al., 2014).

Regarding seasonal influences on psychrotolerant spore counts, we detected a marked decrease in summer and early autumn (July to September). Despite large variations between individual samples, these differences were significant for August and September in contrast to March, December, and January (*p* < 0.05). For other months that showed no significance, the trend was still confirmed in two consecutive years of comparative analyses and was further supported by the missing seasonality of mesophilic spores obtained from identical bulk-tank-milk samples. To our knowledge, only three studies so far have investigated the seasonal effects on psychrotolerant spore counts with conflicting results (McKinnon and Pettipher, 1983; Sutherland and Murdoch, 1994; McGuiggan et al., 2002). McKinnon and Pettipher (1983) analyzed the concentration of psychrotolerant spores in the bulk tanks of four farms and found no significant differences in between summer and winter. McGuiggan et al. (2002) likewise reported that fluctuating spore counts in bulked milk from one processing plant were related neither to meteorological temperature nor to relative humidity. In contrast, Sutherland and Murdoch (1994) detected psychrotolerant spores exclusively in late summer and autumn, when counts in our study were especially low. These contradictory findings may result from the large variety of contamination sources that, depending for example on farm management practices, influence the composition of the milk microbiota to varying degrees. This influence may have changed over time due to altered farm management practices and for the same reason may also depend on the geographical region. The main sources of spores when housing cows are the bedding material (Magnusson et al., 2007), but also the feed (Te Giffel et al., 2002; Vissers et al., 2007). Whenever spores are present in high numbers, for instance in the silo, they are excreted in the feces and may contaminate the teat surface (Magnusson et al., 2007). Elevated spore levels in winter and spring may result from lower temperatures that inhibit the proliferation of accompanying bacteria and thereby promote the growth of PSF during the storage of bedding material or feed. The fact that we found identical predominant species of psychrotolerant spores independently of the season further suggests that they originate from the same source and only reach higher counts due to improved growth conditions.

Biodiversity analyses of 2634 isolates from 28 bulk-tank-milk samples revealed a broad biodiversity of PSF. Of nine genera that were detected, *Paenibacillus* and *Bacillus* were most frequently isolated. This is in line with previous studies that described these two genera as the most important dairy-associated sporeformers (Coorevits et al., 2008; Ivy et al., 2012; Masiello et al., 2014). At species level, the clear dominance of a single group, namely *P. amylolyticus/xylanexedens*, was detected. It accounted for more than 45% of all isolates. In comparison, a majority (41 species) of the remaining 52 species contributed less than 1% each. An uneven abundance distribution with several predominant species or genera and a large fraction of rare bacteria is frequently described in biodiversity analyses (Quigley et al., 2013; von Neubeck et al., 2015). In the context of dairy-associated psychrotolerant sporeformers for example, Ivy et al. (2012) found that six different species (*B. cereus* sensu lato, *Bacillus licheniformis, B. pumilus, P. odorifer, P. amylolyticus/xylanexedens*, and *Paenibacillus graminis*) represent more than 80% of isolates. The clear dominance of one species to the extent revealed in this study, however, is striking and clearly demonstrates its adaptation to the farm and/or dairy environment and its ability to proliferate under the given conditions. Almost 14% of all isolates were affiliated with presumptive novel species (7%) or even novel genera (3%). This high percentage is in line with findings from previous biodiversity studies (Hantsis-Zacharov and Halpern, 2007; Fricker et al., 2011; Ivy et al., 2012; von Neubeck et al., 2015). Here the authors concluded that the milk microbiota is still underexplored. This is supported by the fact that two of the presumptive novel species, *Paenibacillus* sp. nov. 1 and *Paenibacillus* sp. nov. 2 were among the seven most abundant isolates and are not yet described despite their potential technological relevance. *B. cereus* sensu lato, on the other hand, has been reported to be among the most important PSF in bulk tank milk (Vithanage et al., 2016). In this study though, it accounted for merely 0.6% of all isolates thereby indicating only minor relevance.

### Points of Entry of Psychrotolerant Spore-Forming Bacteria into ESL Milk

*Bacillus cereus* sensu lato was the most important sporeformer in ESL milk. It was isolated from more than half of all positively tested packages and batches and accounted for one third of all isolates from end products. However among bulk tank milk isolates, *B. cereus* sensu lato represented merely 0.6%. In contrast, the third most frequently found species (*Paenibacillus* sp. nov. 1) was almost equally prevalent in end products and bulk tank milk (**Figure 5**). It is tempting to assume that both species may have different points of entry into the process chain.

The occurrence of *Paenibacillus* sp. nov. 1 in bulk tank milk and end product was tested to be stochastically dependent (chi-squared), meaning that the prevalence in ESL milk is associated to the prevalence in bulk tank milk. This confirms the expectation that PSF are found in the end product only to the degree they are not removed from bulk tank milk during processing. *P. amylolyticus/xylanexedens* is by far the most prevalent and frequent species in bulk tank milk, but it is not transmitted at all to the end product. This is clear evidence for the high efficiency of spore retention during production, eliminating at least 3.7 log units as determined in this study. For the six batches of ESL milk that were positive for PSF and for which the corresponding bulk tank milk was available, transmission of identical strains could be shown in only half of the cases. In two of these three cases, bulk tank milks had an elevated spore count and transmission was attested for the dominant species only. In both cases *P. amylolyticus/xylanexedens* appeared in the bulk tank milk to a much lesser extent than on average, which explains its absence in ESL milk. Thus, transmission of spores into the end product does occur, but due to high retention during microfiltration it is only relevant in the processing of bulk tank milk with elevated spore counts.

Recontamination along the process chain is likely for species with large discrepancies between abundance in raw and ESL milk, such as *B. cereus* sensu lato and *P. odorifer* (**Figure 5**). This was confirmed by the Pearson’s chi-squared test, which demonstrates that occurrence of both species in bulk tank and ESL milk is stochastically independent (*p* < 0.01). One reason for this is certainly their high prevalence in one production batch from dairy C, which these two species spoiled to 100%. As the bulk tank milk contained only 0.11 MPN/mL and *P. amylolyticus/xylanexedens* was clearly dominant but absent in the end product, *B. cereus* sensu lato and *P. odorifer* most probably entered the product by recontamination. There are plenty of studies reporting on different recontamination sites and the resistance of strains to cleaning and sanitizing conditions. Te Giffel et al. (1997), Faille et al. (2001), and Shaheen et al. (2010) demonstrated that spores of *B. cereus* can adhere to the surfaces of plant equipment and cleaning does not eliminate them. Svensson et al. (2004) isolated identical strains of *B. cereus* from silo tanks over a period of several months and concluded that this was an established in-house flora. Salustiano et al. (2009) was able to relate strains isolated from the surface of the silo tank and filling machine collected after sanitation to strains in the end products. Particular recontamination sites were not analyzed during our study and typing of several strains isolated from ESL milk of the same dairy did not point to persistent recontamination flora. Nevertheless, it is evident from the literature that recontamination is a widespread phenomenon and we consider this to be a probable reason for the large abundance of *B. cereus* sensu lato and *P. odorifer* in the end products of our study.

The counts in the bacterial groups with the highest spoilage potential need to be reduced to improve the milk’s keeping quality. Concerning Gram-negative bacteria, which still represent an important proportion in spoiled ESL milk products, PPR must be avoided. As high PSF counts in bulk tank milk may lead to a transfer of spores into the end product, spore counts in bulk tank milk need to be reduced at the farm level. However, recontamination with PSF is also a major factor and needs to be addressed just like PPR with Gram-negative bacteria by optimizing hygienic conditions in pipes, tanks and the filling process. Aseptic production lines may largely facilitate this task, although accompanied by higher costs.

## Conclusion

Spore counts in raw milk are low and spores are efficiently reduced during ESL milk production. We therefore conclude that the shelf life of ESL milk is determined by raw-milk-associated factors only to a minor extent. In contrast, both recontamination by spores along the production chain, particularly from the *B. cereus* complex, and post-processing contamination with Gram-negative bacteria are important factors leading to premature spoilage. To improve the shelf life of ESL milk, special attention needs to be paid to plant sanitation and disinfection with particular emphasis on eliminating spores.

## Author Contributions

MW and SS conceived and designed the study. ED carried out the laboratory work. ED and MW analyzed and interpreted the data and wrote the manuscript. All authors critically revised and approved the final manuscript.

## Conflict of Interest Statement

The authors declare that the research was conducted in the absence of any commercial or financial relationships that could be construed as a potential conflict of interest.
