# Supplementary material for: Spoilage of Microfiltered and Pasteurized Extended Shelf Life Milk Is Mainly Induced by Psychrotolerant Spore-Forming Bacteria that often Originate from Recontamination
Source: Front Microbiol. 2017 Jan 31;8:135. doi: 10.3389/fmicb.2017.00135 (PMC5281617; doi:10.3389/fmicb.2017.00135)
Supplement: Supplementary file 1 [file Data_Sheet_1.docx]

Supplementary table S1: Abundance distribution of psychrotolerant spore-forming bacteria in 28 bulk-tank-milk samples at species level (n=2634).

| No. | Species | Abundance |
| --- | --- | --- |
| 1 | *Paenibacillus amylolyticus/xylanexedens* | 47.30% |
| 2 | *Paenibacillus odorifer* | 9.72% |
| 3 | *Paenibacillus taichungensis/tundrae* | 7.63% |
| 4 | *Paenibacillus* sp. nov. 1 | 6.57% |
| 5 | *Bacillus pumilus/safensis* | 6.53% |
| 6 | *Paenibacillus taohuashanense* | 3.42% |
| 7 | *Paenibacillus* sp. nov. 2 | 3.15% |
| 8 | *Bacillus simplex* | 2.51% |
| 9 | *Paenibacillus anaericanus* | 2.09% |
| 10 | *Sporosarcina* sp. nov. 1 | 1.52% |
| 11 | *Viridibacillus arenosi/arvi* | 1.33% |
| 12 | *Sporosarcina newyorkensis* | 1.21% |
| 13 | *Lysinibacillus parviboronicapiens* | 0.68% |
| 14 | *Bacillus cereus* sensu lato | 0.61% |
| 15 | *Paenibacillus terrae* | 0.61% |
| 16 | *Virgibacillus* sp. nov. | 0.61% |
| 17 | *Paenibacillus xylanilyticus* | 0.57% |
| 18 | *Paenibacillus oceanisediminis* | 0.49% |
| 19 | *Paenibacillus* sp. nov. 3 | 0.46% |
| 20 | *Paenibacillus lautus* | 0.38% |
| 21 | Gen. nov. 1 | 0.34% |
| 22 | *Paenibacillus sp.* nov. 4 | 0.27% |
| 23 | *Bacillus plakortidis* | 0.23% |
| 24 | *Bacillus licheniformis* | 0.11% |
| 25 | *Filibacter limicola* | 0.11% |
| 26 | *Paenibacillus sp. nov. 5* | 0.11% |
| 27 | *Paenibacillus sp. nov. 6* | 0.11% |
| 28 | *Paenibacillus tibetensis* | 0.08% |
| 29 | *Bacillus psychrosaccharolyticus* | 0.08% |
| 30 | Gen. nov. 2 | 0.08% |
| 31 | *Paenibacillus barcinonensis* | 0.08% |
| 32 | *Paenibacillus borealis* | 0.08% |
| 33 | *Paenibacillus* sp. nov. 7 | 0.08% |
| 34 | *Paenibacillus* sp. nov. 8 | 0.08% |
| 35 | *Sporosarcina* sp. nov. 2 | 0.08% |
| 36 | *Sporosarcina ureae* | 0.08% |
| 37 | *Paenibacillus dongdonensis* | 0.04% |
| 38 | *Bacillus drentenis* | 0.04% |
| 39 | *Bacillus oleronius* | 0.04% |
| 40 | *Bacillus subtilis/tequilensis* | 0.04% |
| 41 | *Cohnella* sp. nov. | 0.04% |
| 42 | Gen. nov. 3 | 0.04% |
| 43 | *Paenibacillus graminis* | 0.04% |
| 44 | *Paenibacillus illinoisensis* | 0.04% |
| 45 | *Paenibacillus lactis* | 0.04% |
| 46 | *Paenibacillus* sp. nov. 9 | 0.04% |
| 47 | *Paenibacillus* sp. nov. 10 | 0.04% |
| 48 | *Paenibacillus* sp. nov. 11 | 0.04% |
| 49 | *Paenibacillus* sp. nov. 12 | 0.04% |
| 50 | *Paenibacillus* sp. nov. 13 | 0.04% |
| 51 | *Psychrobacillus* sp. nov. | 0.04% |
| 52 | *Sporosarcina luteola* | 0.04% |
| 53 | *Virgibacillus carmonensis/necropolis* | 0.04% |


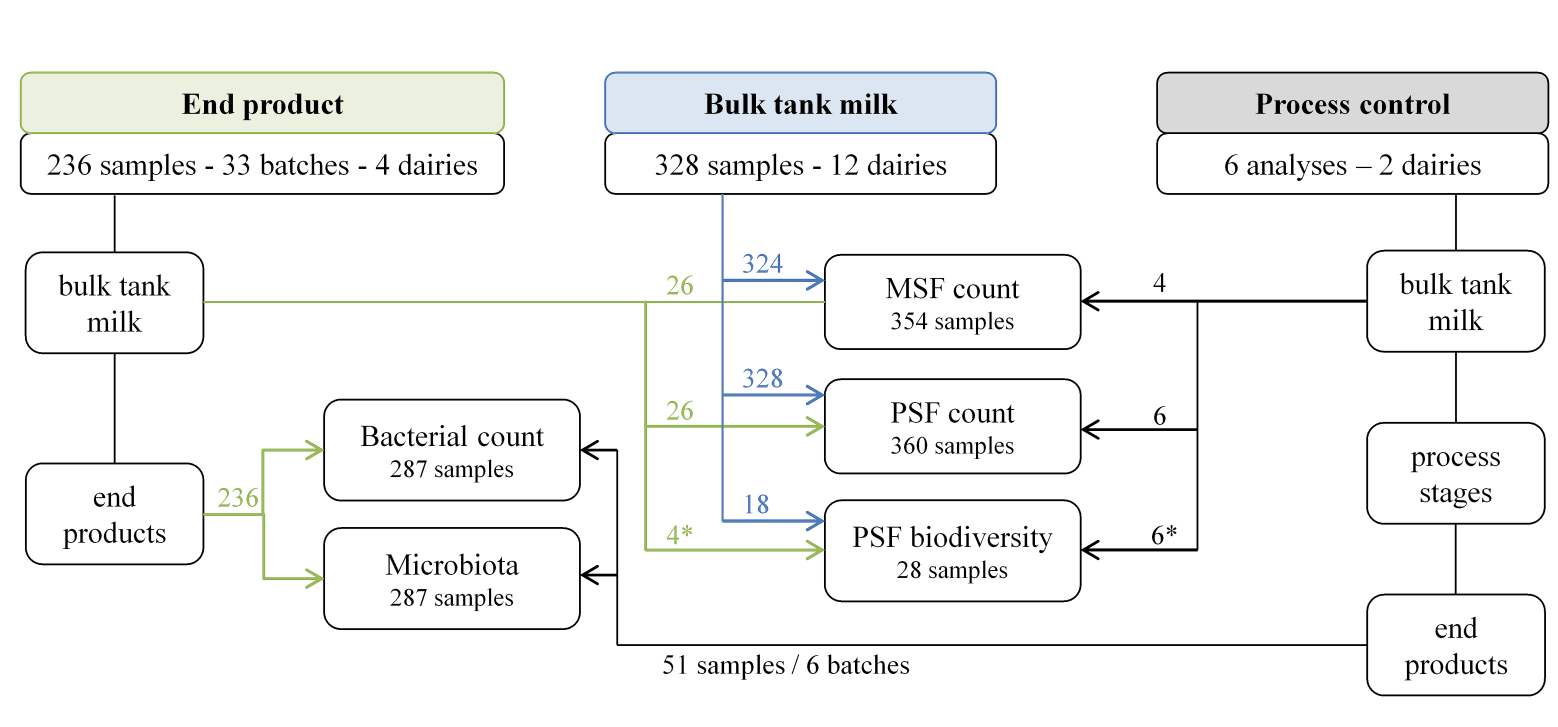


Supplementary figure S1: Study design and sampling, showing the overlap of different study parts. Numbers on the arrows indicate the quantity of samples that were subjected to the corresponding analysis. MSF: mesophilic spore-forming bacteria; PSF: psychrotolerant spore-forming bacteria;*90 isolates of these production batches were typed by RAPD-PCR.


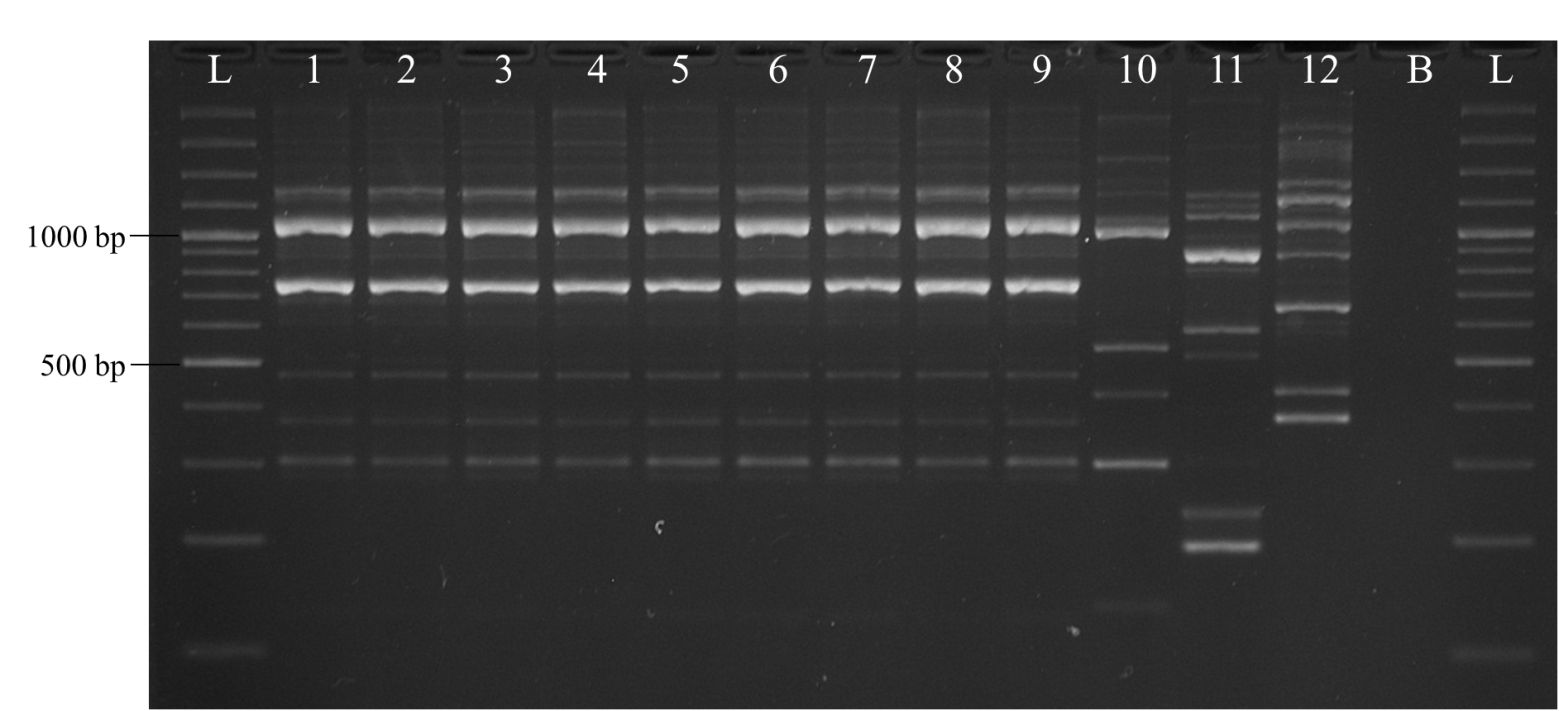


Supplementary figure S2: Band patterns obtained by RAPD typing of twelve isolates of *Paenibacillus* sp. nov. 1, using primer OPA7. Lines 1–9 show identical strains that were isolated from bulk tank milk (lines 1–7) and end products (lines 8–9) of one batch, resulting in identical band patterns with an identical number and intensity of bands. Lines 10–12 show three additional, different strains of the same species, isolated from three other bulk-tank-milk samples and resulting in discriminative patterns. L: ladder; B: blank


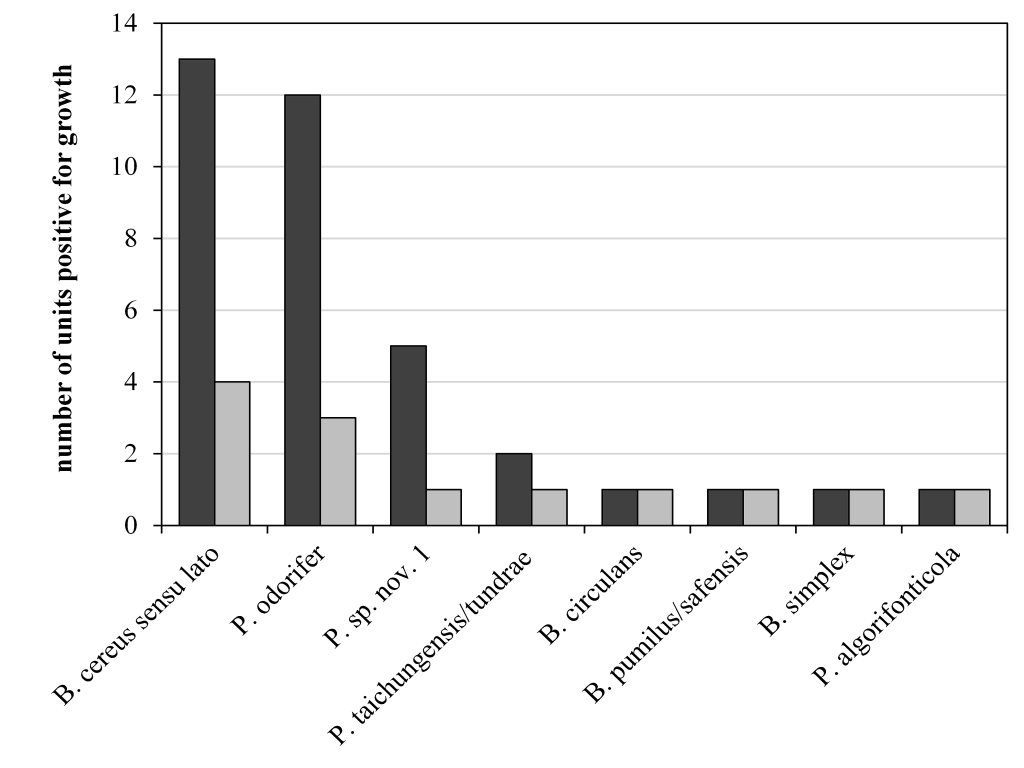


Supplementary figure S3: Species of psychrotolerant spore-forming bacteria isolated from 25 packages of 7 batches of ESL milk at the end of shelf life after storage at 8 °C (n=36). black bars: number of positive packages; gray bars: number of positive batches
